# Aspartate Decarboxylase is Required for a Normal Pupa Pigmentation Pattern in the Silkworm, *Bombyx mori*

**DOI:** 10.1038/srep10885

**Published:** 2015-06-16

**Authors:** Fangyin Dai, Liang Qiao, Cun Cao, Xiaofan Liu, Xiaoling Tong, Songzhen He, Hai Hu, Li Zhang, Songyuan Wu, Duan Tan, Zhonghuai Xiang, Cheng Lu

**Affiliations:** 1 State Key Laboratory of Silkworm Genome Biology, College of Biotechnology, Southwest University, Chongqing, 400716, China; 2 Key Laboratory for Sericulture Functional Genomics and Biotechnology of Agricultural Ministry, Southwest University, Chongqing 400716, China; 3 Institute of Entomology and Molecular Biology, College of Life Sciences, Chongqing Normal University, Chongqing 401331, China

## Abstract

The pigmentation pattern of *Lepidoptera* varies greatly in different development stages. To date, the effects of key genes in the melanin metabolism pathway on larval and adult body color are distinct, yet the effects on pupal pigmentation remains unclear. In the silkworm, *Bombyx mori*, the *black pupa* (*bp*) mutant is only specifically melanized at the pupal stage. Using positional cloning, we found that a mutation in the *Aspartate decarboxylase* gene (*BmADC*) is causative in the *bp* mutant. In the *bp* mutant, a SINE-like transposon with a length of 493 *bp* was detected ~2.2 kb upstream of the transcriptional start site of *BmADC*. This insertion causes a sharp reduction in *BmADC* transcript levels in *bp* mutants, leading to deficiency of β-alanine and N-β-alanyl dopamine (NBAD), but accumulation of dopamine. Following injection of β-alanine into *bp* mutants, the color pattern was reverted that of the wild-type silkworms. Additionally, melanic pupae resulting from knock-down of *BmADC* in the wild-type strain were obtained. These findings show that *BmADC* plays a crucial role in melanin metabolism and in the pigmentation pattern of the silkworm pupal stage. Finally, this study contributes to a better understanding of pupa pigmentation patterns in *Lepidoptera*.

The melanin metabolism pathway is involved in insect cuticle pigmentation and plays an important role in ecological adaption, such as in escaping predation, mimicry, sexual selection, signaling, and thermo-regulation^1^,^2^,^3^,^4^. In insect melanin metabolism, Dopamine is a key pigmentation precursor, and represents a key regulator of color patterns, and its availability is determined by the expression of melanin metabolism genes in this pathway^5^,^6^,^7^. The up-regulated expression of genes encoding rate-limiting enzymes for melanin metabolism, *TH* (*Tyrosine Hydroxylase* gene) and *DDC* (*Dopa Decarboxylase* gene), can catalyze the synthesis of Dopamine and result in Dopamine accumulation, which allows melanin to be created in insects^8^,^9^,^10^,^11^,^12^. By contrast, down-regulated expression and the defective function of insect arylalkylamine-N-acetyl transferases (iaaNATs), which can catalyze dopamine to colorless N-acetyl dopamine (NADA) reactions, can also result in melanism^13^,^14^,^15^,^16^. Furthermore, β-alanine, catalyzed by N-β-alanyl-dopamine synthase (EBONY), can react with dopamine to produce the yellowish compound N-β-alanyl dopamine (NBAD) to create another rate-limited branch for dopamine consumption, which also shows a close relationship with Dopamine accumulation^17^,^18^,^19^. Many insect melanic mutants that result from Dopamine accumulation have been found to be associated with β-alanine deficiency, and β-alanine treatment can restore these mutants to wild-type phenotypes^20^,^21^,^22^,^23^,^24^,^25^,^26^. Another study found that the adult *black* mutant of *Tribolium castaneum* is a type of β-alanine deficiency melanic mutant that resulted from the marked down-regulation of Aspartate decarboxylase (ADC), which can catalyze aspartic acid to β-alanine^27^. The *ADC* genes (also known as *black*) have been identified in several *Lepidoptera* insects; however, no direct phenotypic evidence has been reported to indicate that ADC participates in pigmentation^28^,^29^,^30^,^31^,^32^.

There are more than 100 body color mutants that have been reported in silkworms^33^,^34^. Based on research into the molecular mechanism for a number of body color mutants, the major composition and corresponding regulatory relationships involved in the silkworm melanin metabolism pathway have become increasingly clear, which makes the silkworm a model species for the study of the molecular mechanisms of pigmentation in *Lepidoptera*^13^,^14^,^16^,^28^,^35^,^36^,^37^,^38^.

In the pupae metamorphosis period of silkworms, normal pupae exhibit an amber body color. If the function of *Bmebony* is abolished during this period, Dopamine will accumulate excessively because it cannot be converted to NBAD, which will makes the pupae adopt a melanism phenotype, which is the cause of the *sooty* (26-0.0) mutant^36^. The mutant *melanism* (18-41.5) is another body color mutant in which melanism is expressed in the late pupal–moth stage^13^,^14^,^33^,^34^. This occurs because of the dysfunction of Bm-iaaNAT, which cannot catalyze the conversion step from dopamine to NADA in order to accumulate Dopamine excessively in order to make melanism^13^,^14^,^16^. In addition to these two mutants described above, a unique melanic pupa mutant, *black pupa* (*bp*), showed melanization specifically in the pupal stage^33^,^34^. The genetic locus of *bp* (11-42.5) is independent of the *sooty* and *melanism* loci, and we speculate that there is other unknown pivotal gene that determines pupa pigmentation. Using positional cloning, gene expression analysis, RNAi, and biochemical and physiological analysis, we identified the *Bombyx mori* gene *Aspartate gecarboxylase* (*BmADC*) that is responsible for the mutant phenotype. *BmADC* shows low expression in the pupa stage of the *bp* mutant, which reduces β-alanine content. NBAD synthesis is hindered, which subsequently leads to an accumulation of excess dopamine, and eventually results in the melanism phenotype. To the best of our knowledge, the *bp* mutant is the first phenotype that shows the role of ADC in melanin metabolism in *Lepidoptera*. Our study should provide a theoretical basis to understand the role that ADC plays in this pigmentation model of Lepidoptera insect pupae metamorphosis, and also represents an important contribution to body pigmentation research.

## Methods

### Silkworm Strains

The *bp* mutant strain 16-100 (*bp*/*bp*) and three wild-type strains, Dazao (+^*bp*^/+^*bp*^), C108 (+^*bp*^/+^*bp*^), and N4 (+^*bp*^/+^*bp*^), were obtained from the silkworm gene bank at Southwest University and were reared on fresh mulberry leaves under a 12 hr/12 hr light/dark photoperiod at 24 °C.

### Chemicals

β-alanine (A9920) and Dopamine (H8502) were purchased from Sigma. NBAD standards were provided by Professor Michael R. Kanost and Dr. Neal T. Dittmer (Department of Biochemistry, 141 Chalmers Hall, Kansas State University, Manhattan, KS 66506-0116, USA). The β-alanine and aspartate standards used for quantitative analysis were provided by the Institute of Animal Nutrition, Sichuan Agricultural University (Chengdu, Sichuan, China).

### Mapping of *bp* locus

Two silkworm strains, C108 (+^*bp*^/+^*bp*^) and 16–100 (*bp*/*bp*) were selected for genetic mapping. F_1_ offspring were produced from a cross between a female C108 and a male *bp*. 20 BC_1_F, and progeny from the cross (C108 × 16–100)♀ × 16-100♂ were used for linkage analysis, while 562 BC_1_M progeny from the cross 16-100♀ × (C108 × 16-100)♂ were used for recombination analysis. Polymorphic PCR markers (the SSR markers of 11^th^ linkage group^39^ and several markers designed by ourselves) were identified among the parents, and these were assessed in BC_1_F individuals. In BC_1_M progeny, individuals exhibiting a normal amber body color (heterozygosis for the *bp* locus) and melanic body color (homozygosis on *bp* locus) were genotyped. Primers used for genotyping are listed in Table S1.

### Cloning of *BmADC* cDNA

Total RNA was extracted from Dazao and 16–100 at 6 h of pupation. The full-length cDNA sequence of the *BmADC* gene was obtained by rapid amplification of cDNA ends (RACE) using the GeneRacer kit (Invitrogen) according to the manufacturer’s protocol. The primers used for full-length cloning of *BmADC* are listed in Table S1. PCR products were cloned into a PMD19-T vector (Takara) and sequenced. The accession numbers of *BmADC* cDNA in Dazao and 16–100 were KM523624 and KM523625, respectively. All sequence determinations were performed on three biological replicates per sample.

### Homology and phylogenetic analyses

The ADC homology was searched by BlastP at the NCBI website (http://www.ncbi.nlm.nih.gov/), flybase (http://flybase.org/), bettlebase (http://beetlebase.org/), silkDB (http://www.silkdb.org/silkdb/), MonarchBase (http://monarchbase.umassmed.edu/), and Manduca base (http://agripestbase.org/manduca/). Additionally, the amino acid decarboxylase with a Pyridoxal Phosphate domain in *Drosophila melanogaster*, *B. mori*, and *Tribolium castaneum* were also searched using the BlastP and tBlastN programs. Using the online Muscle program (http://www.ebi.ac.uk/Tools/msa/muscle/) with defult settings, the amino acid sequences were aligned. Subsequently, the neighbor-joining method in MEGA4^40^ was used to construct a phylogenetic tree, and Bootstrap vaules were obtained based on 1000 bootstrap replications.

### Genomics PCR and RT-PCR

Primers were designed primers based on the silkworm 9x assembly genome database to detect differences in the upstream sequences of the transcriptional start site for the *BmADC* gene between Dazao (wild-type) and 16–100 (*bp* mutant). The PCR products were cloned into a PMD19-T vector (Takara) and sequenced (three biological replicates for each sample). The characteristics of each sequence were analyzed using BmTEdb (http://gene.cqu.edu.cn/BmTEdb/) and genomatix (http://www.genomatix.de/). RT-PCR was performed to analyze the expression patterns of *BmADC* in the wild-type strains. Total RNA was extracted and purified from the silkworm whole body for several developmental stages (from the wandering stage to eclosion of adults) using TRIzol (Invitrogen) according to the manufacuturer’s protocol, and was subjected to cDNA synthesis using oligo (dT) primer and MLV reverse transcriptase provided in the Promega P1300 kit (Promega; total RNAs at each development point were from three individuals or three replicates each developmental point). Primers designed for RT-PCR are listed in Table S1. The *BmActin3* gene was used as an internal control.

### Quantitative RT-PCR

Quantitative RT-PCR was performed to measure the expression levels of *BmADC* in both the Dazao and 16–100 strains using the ABI Prism 7000 sequence detection system (Applied Biosystems, Foster City, CA, USA) with a SYBR Premix Ex-Taq kit (Takara) according to the manufacturer’s protocol. The primers designed for qRT-PCR are listed in Table S1. The *B. mori* gene *Eukaryotic translation initiation factor 4A* (microarray probe ID: sw22934) was used as an internal control. All assessments were performed on three biological replicates per sample.

### RNAi of *BmADC*

RNAi was performed to validate the function of the *BmADC* gene. The ds*BmADC* and ds*Red* (red fluorescent protein, used as control) targeting oligos were synthesized using the T7 RiboMAXTM Express RNAi System (Promega). Then, dsRNA was diluted to 12 μg/μl, and a dose of 120 μg was administered to each individual. The injection time was selected according to the temporal expression patterns of the *BmADC* gene 48 h after wandering. Subsequently, observation and qRT-PCR analysis of gene expression were carried out at day 1 of pupation, and the tissues for gene expression analysis after RNAi were selected from the parts with a distinct phenotype difference between the interference individuals and control individuals (three biological replicates for each sample). The non-diapause strain N4 (wild-type) was selected for RNAi. The statistical analyses of the RNAi experiments are listed in Table S2. Primers used for RNAi experiments are listed in Table S1.

### Quantification of amino acids and catecholamines in wild-type and *bp* mutant silkworms

Pupae were selected at 0 h of pupation of Dazao and 16–100 for amino acids and catecholamine content analysis (each sample was carried out in triplicate biological replicates, and each sample contained a mixture of three pupae). The free amino acids were extracted as follows: samples were homogenized with 1 ml 0.1 M HCl in centrifuge tubes, then were dissociated from the homogenate for 15 min using ultrasonication followed by centrifugation at 12,000 rpm and 4 °C. The supernatants (600 μl) were transferred to new tubes containing 600 μl 10% sulfosalicylic acid, and were centrifuged one more time at 12,000 rpm and 4 °C for 15 min. The supernatants were transferred and filtered through 0.22 μm membranes. The free amino acids were quantified according to the Le Boucher method^41^. A Hitachi L-8800 Amino Acid Analyzer Physiological fluid system (a lithium system; Tokyo, Japan) was used for amino acid content analysis. The extraction and quantification of Dopamine and NBAD were performed according to Koch’s method^18^. An Agilent 1260 Infinity High-Performance Liquid Chromatography analyser and Symmetry Shield RP18 columns (5 μm, 4.6 × 250 mm, Waters) were used for HPLC analysis. Amino acids and catecholamine standards were identified based on retention times as follows: aspartate, 11.76 min; β-alanine, 59.27 min; Dopamine, 8.065 min; and NBAD, 18.404 min (Fig. S2A and S2B). Each sample, which was mixed from two pupae, was analyzed in triplicate biological replicates.

### β-alanine treatment

The *bp* mutant strain 16–100 was selected for β-alanine (dissolved in 0.75% saline) injection at 60 h after wandering, and the dose was 400 μg per pupa. Mutant pupae injected with 0.75% saline were used as controls. After injection, a pupae phenotype was observed and the expression of *BmDDC* and *Bmebony* were measured at 6 h of pupation. The efficiency of β-alanine treatment is listed in Table S3.

## Results

### *BmADC*, a gene in the melanin metabolism pathway, is located in the mapping region

**Figure 1:**
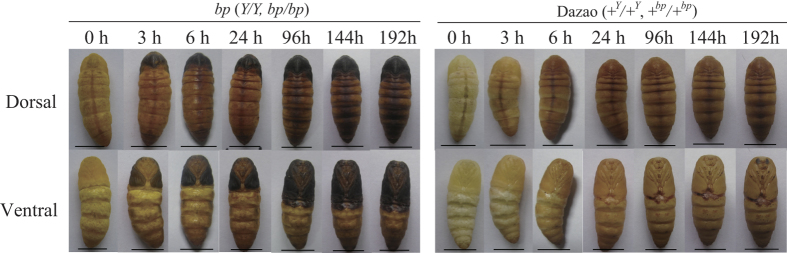
Phenotype of Dazao (wild-type) and 16–100 (*bp* mutant) at 0, 3, 6, 24, 96, 144, and 192 h of pupation under 24 °C . *Y* represents *yellow blood* locus (2–28.6) in the mutant strain. Scale bar: 1 cm.

**Figure 2:**
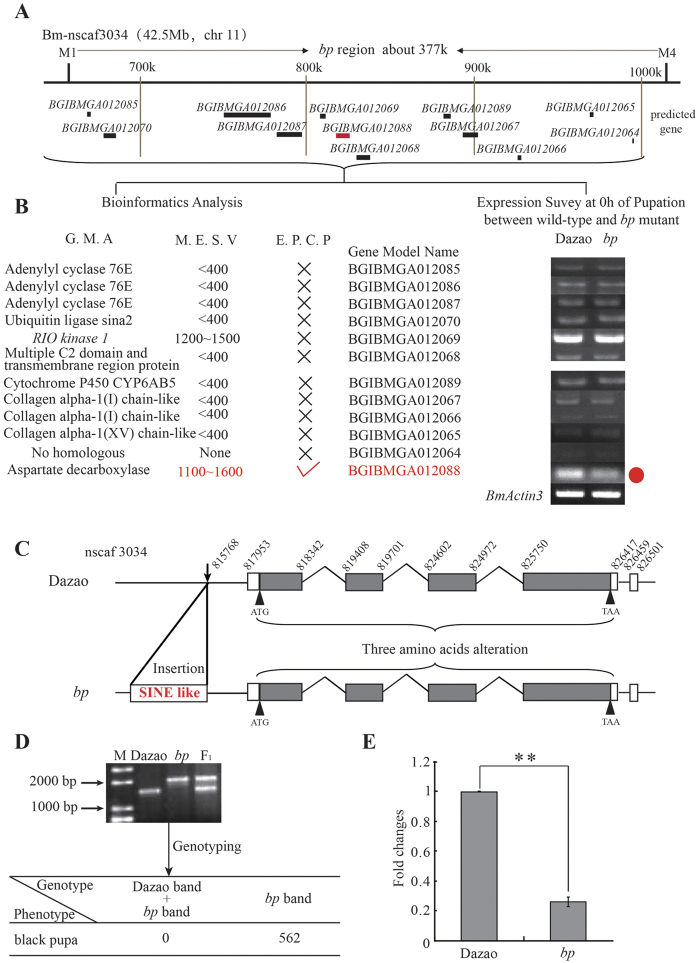
Genetic basis of the *bp* mutant . (**a**) Fine mapping of the *bp* locus. The *bp* locus was narrowed between the PCR markers M1 and M4, a region of ~377 kb. The solid line shows segment predicted gene models, and *BGIBMGA012088* is marked in red. (**b**) Candidate genes in the 377 kb region. GMA, MESV, and EPCP represent Gene Mode Anotation, The Microarray Expression Signal Value (at 72 h of wandering or 0 h of pupation), and Evidence of Participating in Cuticle Pigmentation, respectively. The values of the Microarray Expression Signal less than 400 indicate that gene expression abundance is especially deficient^53^. The forked symbol represents deficiency of unambiguous evidence, whereas the red hook symbol represents existing unambiguous evidence. The red solid cycle represents an obvious expression difference of *BGIBMGA012088* between wild-type and *bp* mutant silkworms. (**c**) Sequence differences of *BmADC* between wild-type and *bp* mutant silkworms. (**d**) Genotyping analysis between G1 (polymorphism marker) and the *stony* locus. M represents a DNA marker. (**e**) Relative expression levels of *BmADC* between wide-type and *bp* at 24 h of pupation under 24 °C. (Student’s *t*-test; n=3; **, p < 0.01). Data are presented as means ± SD.

The pigmentation of wild-type pupae begins at 3 h of pupation, and they exhibit an amber body color throughout metamorphosis (Fig. 1). Compared to wild-type pupae, this process in the *bp* mutant also begins at 3 h of pupation, yet melanism also occurs (Fig. 1). From 3 to 24 h of pupation, the mutant pupae blacken rapidly, but from 24 h to 8 days of pupation, it reaches a relatively slow melanism stage (Fig. 1). The *bp* locus was mapped within a ~377 kb region that contains 12 predicted gene (according to Bm_nscaf3034; chromosome 11; Fig. 2A). Then, these genes were analyzed and annotated these genes using the silkworm genome database, SilkDB^42^, the BLASTp program hosted by the National Center for Biotechnology Information (http://blast.ncbi.nlm.nih.gov/Blast.cgi), and the Pfam database (http://pfam.xfam.org/) (Fig. 2B). Additionally, the gene expression pattern in both wild-type and *bp* mutant silkworms was determined. Based on these data, we focused on a predicted gene, *BGIBMGA012088*, which was expressed significantly lower in mutant than in wild-type silkworms (Fig. 2B). This gene encodes a typical Pyridoxal Phosphate structural domain. Its orthologs are aspartic acid decarboxylase (ADC) in D. melanogaster and T. castaneum (with a similarity of 62% and 65%, respectively; Fig. S1A). In the D. melanogaster and T. castaneum melanin metabolism pathways, ADC proteins perform a catalytic function for the synthesis of β-alanine, and are involved in melanin metabolism from Dopamine to NBAD^22^,^25^. This process consumes the melanin precursor and converts it to a yellowish pigment precursor, which generates the amber body color. Based on the functional similarity of the orthologous genes, we speculated that the *BGIBMGA012088* gene might also be involved in melanin precursor transformation in the melanin metabolism pathway, and so we named it *BmADC*.

### *BmADC* was closely linked to the *bp* locus and its expression was reduced significantly

The full-length cDNA transcripts of *BmADC* in wild-type (Dazao) and *bp* mutant *B. mori* were cloned. In the wild-type animals, the transcript was 1848 *bp* (without poly A) and contained four exons, encoding a protein of 511 amino acids (Fig. 2C). Compared with the CDS of the wild-type silkworm, 26 single nucleotide substitutions in the ORF and three nucleotide insertions in the 3′-UTR were detected in the *bp* mutant (Fig. 2C). Although this created two amino acid residue changes in the protein product, the predicted tertiary structures of these proteins were not different from that of the wild-type as predicted by Phyre2 (http://www.sbg.bio.ic.ac.uk/phyre2/html/page.cgi?id=index) online analysis (Fig. S1C). The regulatory regions ~2.2 kb upstream of the transcription start sites were analysed and a 493 *bp* insertion was found in the *bp* mutant (Fig. 2C). Based on the insertion, a polymorphic marker G1 was designed and no recombination events were observed between it and the *bp* locus (Fig. 2D). Further analysis in the Bmtedb database showed that the insertion fragment was a short SINE transposon (KM523626). In eukaryotes, robust evidence suggests that transposable elements can regulate gene expression^33^,^43^,^44^. Therefore, we speculate that this insertion might be the reason for the significantly lower *BmADC* gene expression levels. Based on the gene mapping results, the different expression levels of the *BmADC* gene, and the differences in gene expression sequences in gene regulatory regions in the wild-type and *bp* mutant, these findings identify *BmADC* as the causative gene for the *bp* mutant phenotype.

### RNAi for *BmADC* yielded melanic pupae

**Figure 3:**
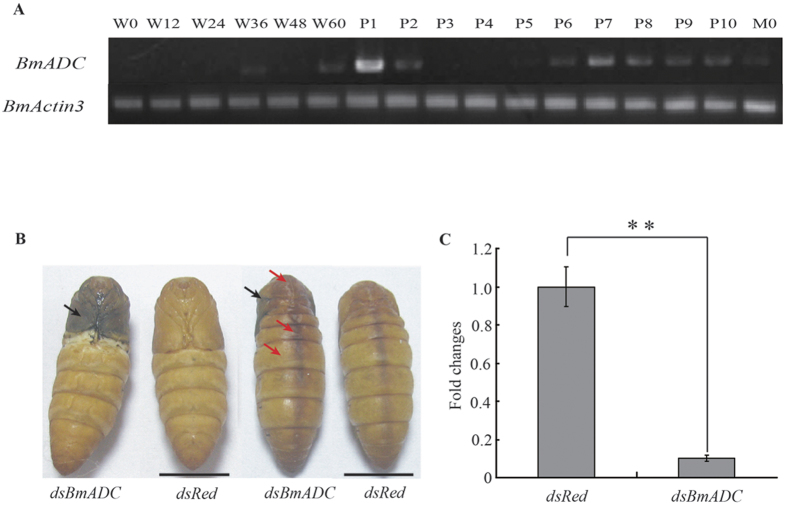
RNAi of *BmADC*. (a) The temporal expression pattern of *BmADC* from the wandering to moth stages. W0-W60 represents 0, 12, 24, 36, 48, and 60 h of the wandering stage, respectively. P1-P10 represents days 1 to 10 after pupation, respectively. M0 represents the first day of the moth stage. *BmActin3* gene was used as an internal control for RT-PCR. (**b**) Phenotype of pupae at day 1 of pupation after RNAi. The black and red arrows point to the melanic and dark areas in the ds*BmADC* injected individuals, respectively. Scale bar: 1 cm. (**c**) Relative expression levels of *BmADC* at day 1 of pupation in individuals subjected to RNAi (n = 3; ds*Red* as a control; Student’s *t*-test, **, p < 0.01). Data are presented as means ± SD.

The expression patterns of *BmADC* during pupal metamorphosis were determined and found that the expression levels of *BmADC* fluctuated during the development period. From 0 to 36 h at wandering, *BmADC* maintained a weak level of expression, and had a high level of expression at the first day of pupation, which is a critical time for pupae pigmentation (Fig. 1). Based on the expression pattern, ds*BmADC* was injected into wild-type larvae at wandering 48 h and the individuals injected with constructs targeting ds*Red* were used as a control. The phenotype was observed at the first day of pupation. The control group was found to maintain a normal color pattern and to display a light amber color, while 56.7% individuals in the interference group showed a significantly darker brown color on the dorsal side compared to the control group (Fig. 3B, Table S2). Notably, the wing of the interference group animals showed a melanism phenotype similar to the *bp* mutant (Fig. 3B), Moreover, qRT- PCR results indicated that the expression level of the corresponding *BmADC* gene was significantly lower in the individuals injected with ds*BmADC* than that in control individuals injected with a dsRed targeting construct (Fig. 3C). These results indicated that the *BmADC* gene was involved in the normal amber color formation of silkworm pupae.

### The absence of β-alanine and excessive accumulation of dopamine in the *bp* mutant

**Figure 4:**
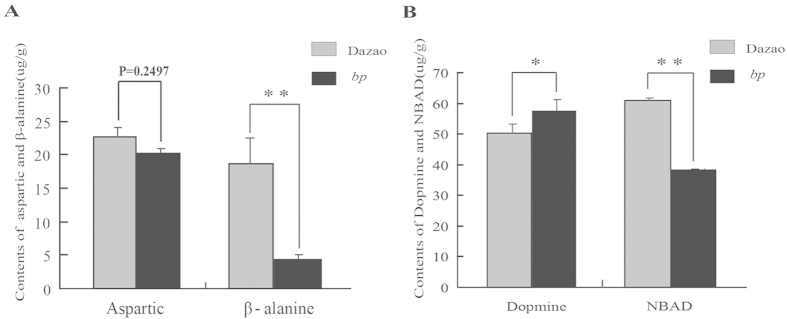
Quantification of Catecholamines and animo acids involved in the melanin metabolism pathway between wide-type and *bp* mutant silkworms . (**a**) Quantification of aspartate and β-alanine between Dazao (wild-type) and 16–100 (*bp* mutant) at 0 h of pupation (Student’s *t*-test; n = 3; **, p < 0.01). Data are presented as means ± SD. (**b**) Quantification of Dopamine and NBAD between Dazao (wild-type) and 16–100 (*bp* mutant) at 0 h of pupation (Student’s *t*-test; n = 3; *, p < 0.05, **, p < 0.01). Data are presented as means ± SD.

In *Tribolium*, aspartic acid decarboxylase catalyzes the conversion from Aspartate to β-alanine that when combined with dopamine can be converted into NBAD by the catalysis of EBONY protein^23^,^24^,^25^. Therefore, reduced *BmADC* expression can result in the insufficient synthesis of β-alanine. This can in turn affect the generation of NBAD and lead to the excessive accumulation of dopamine, which ultimately allows for pupae melanism. To test this hypothesis, the content of related amino acids and catecholamine levels were examined in both the wild-type and *bp* mutant silkworms. No significant difference in aspartic acid, the substrate of ADC protein, was detected between the *bp* mutant and wild-type, while the content of β-alanine, the catalysis product, in *bp* was only 22% of that in wild-type individuals (Fig. 4A). Subsequently, β-alanine deficiency can obstruct transfer from Dopamine to NBAD. Our findings showed that the content of NBAD in the *bp* mutant was only 62% of that in wild-type silkworms, while the dopamine content in the *bp* mutant was ~1.15 fold of that in the wild*-*type silkworms (Fig. 4B). Biochemical analysis showed the mutant phenotype resulted from β-alanine deficiency, which is similar to the *black* mutant in *T. castaneum*, and also confirmed the dysfunction of *BmADC*.

### *bp* mutant reverted to wild-type after β-alanine treatment

**Figure 5:**
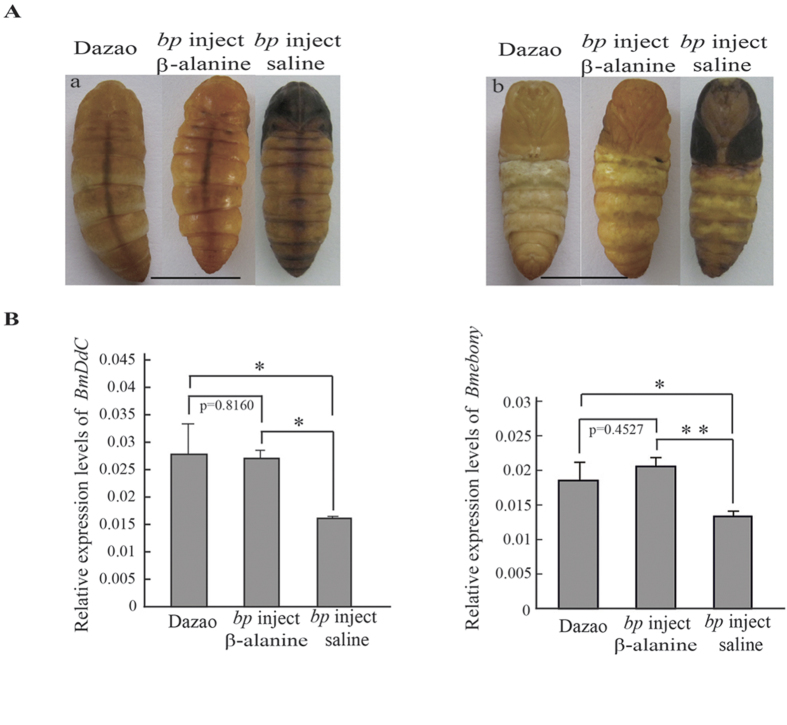
Physiological rescue of the *bp* mutant via β-alanine injection. (**a**) The phenotype of the *bp* mutant after β-alanine injection at 6 h of pupation under 24 °C. a and b represent the dorsal side and ventral side phenotype of the wild-type, *bp* mutant, and β-alanine treated pupae, respectively. Scale bar: 1 cm. (**b**) Relative expression levels of *BmDDC* and *Bmebony* between Dazao (wild-type) and 16–100 (*bp* mutant) at 6 h of pupation (Student’s *t*-test; n = 3; **, p < 0.01). Data are presented as means ± SD.

In *bp* mutants, owing to deficiency of β-alanine, the synthesis of NBDA is restricted, making Dopamine excessively accumulate. Exogenous β-alanine was injected into *bp* mutants. A total of 66.7% of injected individuals reverted to amber, while the control group all exhibited melanism (Fig. 5A, Table S3). The expression level of *BmDDC* was not significantly different between reverted and wild-type individuals, while *bp* individuals were used as the control group; the expression levels of *BmDDC* were significantly lower than those of the other two groups (Fig. 5B). Our previous findings in the silkworm *mln* mutant showed that in the body integument, the accumulated melanin precursor, Dopamine, could inhibit the expression of *BmDDC*. Therefore, in the reverted individuals injected with β-alanine, the up-regulated expression of *BmDDC* showed that the excessive accumulated Dopamine was consumed in these individuals, which is consistent with the reverted amber color. Further investigation of the NBAD synthase gene *Bmebony* showed that its expression level in the reverted individuals was only somewhat higher than that in wild-type individuals, whereas in the *bp* control group, the expression level of *Bmebony* was significantly lower than that of the other two groups (Fig. 5B). This result indicated that, in the reverted group, supplemented exogenous β-alanine might along with the excess accumulation of Dopamine induce the up-regulation of *Bmebony* that would result in the transformation of excess dopamine into the yellow pigment precursor, NBAD, which was coincident with body color reversion.

## Discussion

**Figure 6:**
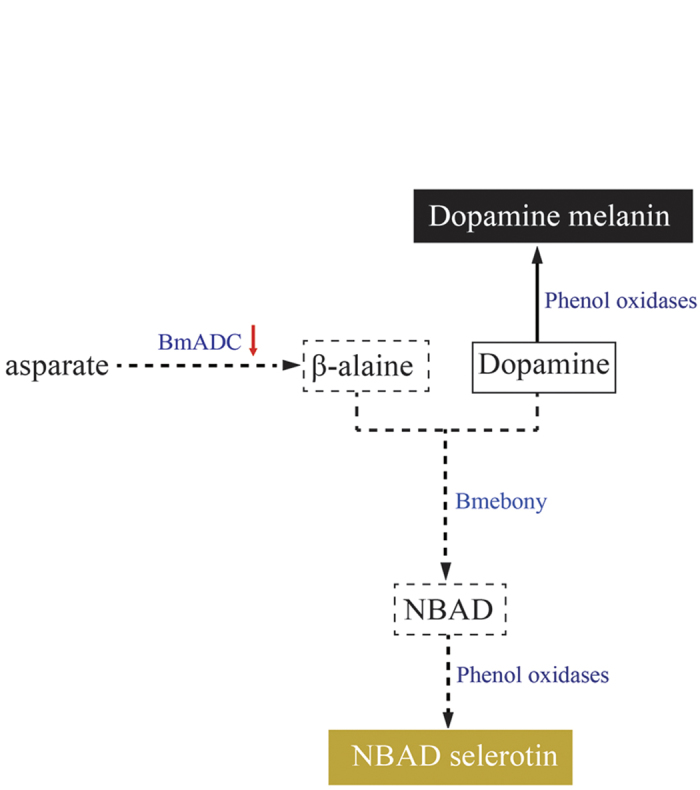
A schematic overview of the phenotype of the *black pupa* mutant . Enzymes are shown in blue. The red arrow represents the expression level of *BmADC* that was reduced sharply in the *bp* mutant. The dotted arrows indicated attenuated biochemical reactions. The solid arrow represents an intensive biochemical reaction. The solid and dotted frames represent the accumulation and insufficiency of biochemical components, respectively. The black box represents the color of Dopamine melanin. The yellowish box represents the color of NBAD selerotin.

In this study, we speculated the causative mechanism of the *bp* mutant based on a series of findings which are summarized as follows: in the *bp* mutant, expression of *BmADC* decreased markedly in the early pupa stage, resulting in reduced synthesis of β-alanine. Then, NBAD synthesis was blocked, which reduces Dopamine consumption and results in excessive accumulation, resulting in melanic pupae (Fig. 6).

There are two coexisting β-alanine synthesis pathways in insects, asparagic acid decarboxylation and uracil hydrolysis. ADC and Dihydropyrimidine dehydrogenase (DPYD) are the respective critical rate-limiting enzymes among the two mentioned pathways^17^,^25^,^29^,^45^,^46^. The temporal expression pattern of these two genes in Dazao showed that *BmDYPD* had high expression in the larval stage, which was not the stage for *BmADC* high expression (Fig. S4A and S4B). We speculate that the synthesis of β-alanine principally depends on the uracil pathway in the larval stage and that the down-regulation of *BmADC* in this stage will not affect β-alanine synthesis, so it will not account for the *bp* larval melanism (Fig. S3A). However, *BmADC* has a much higher level of expression than that of the *BmDYPD* in the 1^st^ day of pupation, which is the most critical stage for pupa pigmentation. Simultaneously, the lower expression of *BmADC* did affect the content of β-alanine, which indicates that most β-alanine in this stage might be synthesized by the asparagic acid decarboxylation pathway. Therefore, if the expression of the *BmADC* is significantly down-regulated at this stage, β-alanine synthesis will be obstructed, which will subsequently affect the pupae pigmentation pattern and delay melanism (Fig. 1, Fig. S4). Subsequently, from the 2^nd^ to 10^th^ day of pupation of the wild-type silkworms (for the body color, there was no significant difference between the two stages), the expression of *BmADC* was reduced remarkably (Fig. S4B), and the expression level of *BmDYPD* was also at a low level (Fig. S4B). So, in the wild-type silkworms, we speculate that β-alanine was not the key factor for pupae pigmentation during this stage (Fig. 1). Thus, in this stage, the reduced levels of *BmADC* expression might not markedly change the extent of melanism in the *bp* mutant because β-alanine is not the important factor for pigmentation (Fig. 1). Additionally, the *bp* mutant does not exhibit melanism in the following moth stage (Fig. S3B). Indeed, when we dissected the pupae cuticle of *bp* mutants in the late pupal stage (P9 or P10), no difference between the new moth cuticle was detected in this stage for the *bp* mutant and wild-type silkworms (data not shown). To explore the reason for these findings, we investigated the expression pattern of the NBAD synthetase gene, *Bmebony*, as well as *Bm-iaaNAT*, from the pupal stage to the moth stage in the Dazao strain. We found that the expression of *Bmebony* was significantly down-regulated in the late pupal (P8) and moth (M1) stages, indicating that NBAD synthesis in this stage is less active than in the early stage (Fig. S4B). This finding indicates that less β-alanine is needed to for synthesis of NBAD for pigmentation. Therefore, although the expression of *BmADC* is down-regulated, in *bp* mutants the pigmentation pattern will not be affected by the material deficiency in the moth stage. Additionally, the product of the highly expressed *Bm-iaaNAT* can convert the accumulated dopamine to the colorless NADA, which will not lead the melanized moth (Fig. S4B). Bm-iaaNAT functions normally in the *bp* mutant; thus, accumulated dopamine can also be converted into NADA, which will make no body color difference in the adult between wild-type and *bp* mutant late pupal and moth stages (Fig. S3B). Although several key genes identified in previous studies were found to regulate pigment synthesis and color patterns, the *BmADC* is indispensable for the early pupal stage. Nonetheless, the factors that finely regulate the high expression levels of *BmADC* in the early pupal stage are not clear, and are the subject of our ongoing research.

In addition to the specifically high expression in the early stage of pupation, the expression of *BmADC* is also sensitive to temperature. The expressions levels of *BmADC* under the high and low temperature are both down-regulated compared with the expression levels at room temperature (24 °C; Fig. S5B). Approximately ~6.3 kb regulatory sequences upstream of *BmADC* (from the end of *BGIBMGA012069* ‘s ORF to the transcriptional start site of *BmADC*) were analysed in wild-type and *bp* mutant silkworms via the genomatrix online program, and 11 heat shocking factor binding sites were detected that might be involved in the regulation of *BmADC* expression (Fig. S5A). The lower levels of *BmADC* expression at low temperature are always accompanied with a darker body color of both the wild-type and *bp* mutant at pupation (brown of wild-type and more melanic in *bp*), which further suggests that *BmADC* participates in the pigmentation pattern at the early pupal stage (Fig. 1, Fig. S5B). Simultaneously, we speculated that the expression pattern of *BmADC* at a low temperature might reflect the insect adaptability to the environment (for example, the melanized body color at a low temperature facilitates the heat absorption^2^; the melanic body color can improve resistance of insect to pathogenic microorganism and Ultraviolet A 11,47; or the melanized body color is able to improve silkworm feeding efficiency^48^). Additionally, the expression levels of *BmADC* are also down-regulated under high temperature, but the body color is lighter than that under normal temperature (the body color of *bp* mutant reverts to that of the wild-type). To explain this discrepancy, the possible explanation may be that the higher temperature can induce a much higher activity of *BmADC* to catalyze the synthesis of β-alanine 49,50,51,52, which could be consumed in Dopamine propionylation and result in a lighter body color. This special mode of regulation could make the silkworm regulate body temperature by lightening body color to reduce injury from high temperature, but also act as an efficient resource to conserve amounts of gene expression.

To date, the effects of the *ADC* gene on insect body color have only been reported in *Drosophila* and *Tribolium*^22^,^25^. Additionally, the melanism phenotype caused by ADC functional deficiency presents only in adult stages in both types of insects. Herein, we found that the *ADC* gene can also affect pupa pigmentation, and this is the first report confirming that this gene could be involved in insect pigmentation patterns in *Lepidoptera*. Additionally, the pigmentation pattern in the early pupal stage of many other moths, such as *Manduca sexta* or *Biston betularia*, are similar to that in silkworms, which indicates that ADC might participate in early pupal stage pigmentation and play a role similar to *BmADC* in these species. Overall, not only can our research uncover the molecular mechanism of the silkworm pupal-specific melanism mutant *bp*, which is involved in replenishing the silkworm melanin metabolism pathway, but it also provides a reference for body color pattern research in other insects, especially *Lepidoptera*.

## Additional Information

**How to cite this article**: Dai, F. *et al.* Aspartate Decarboxylase is Required for a Normal Pupa Pigmentation Pattern in the Silkworm, *Bombyx mori*. *Sci. Rep.* **5**, 10885; doi: 10.1038/srep10885 (2015).

## Supplementary Material

Supplementary Information
