# Supplementary material for: Aspartate Decarboxylase is Required for a Normal Pupa Pigmentation Pattern in the Silkworm, Bombyx mori
Source: Sci Rep. 2015 Jun 16;5:10885. doi: 10.1038/srep10885 (PMC4468592; doi:10.1038/srep10885)
Supplement: Supplementary Information [file srep10885-s1.pdf]

Supplementary information

Aspartate Decarboxylase is Required for a Normal Pupa Pigmentation Pattern in the Silkworm, *Bombyx mori*

Fangyin Dai[1, 2 *], Liang Qiao[1, 3*], Cun Cao[1, 2*], Xiaofan Liu [1, 2*], Xiaoling Tong[1, 2], Songzhen He[1, 2], Hai Hu[1, 2], Li Zhang[1, 2], Songyuan Wu[1, 2], Duan Tan[1, 2], Zhonghuai Xiang[1, 2], Cheng Lu[1, 2]

[1]State Key Laboratory of Silkworm Genome Biology, College of Biotechnology, Southwest University, Chongqing, 400716, China

[2]Key Laboratory for Sericulture Functional Genomics and Biotechnology of Agricultural Ministry, Southwest University, Chongqing 400716, China

[3]Institute of Entomology and Molecular Biology, College of Life Sciences, Chongqing Normal University, Chongqing 401331, China

*These authors contributed equally to this work.

To whom correspondence should be addressed: Fangyin Dai, State Key Laboratory of Silkworm Genome Biology, College of Biotechnology, Southwest University, Chongqing,400716 China, Tel: +86-23-68250793, Fax: +86-23-68251128, e-mail address: fydai@swu.edu.cn, or Cheng Lu, State Key Laboratory of Silkworm Genome Biology, Southwest University, Chongqing, China, e-mail address: lucheng@swu.edu.cn.

Running title: Repression of *BmADC* for silkworm *black pupa* mutants

5 Supplementary Figures
3 Supplementary Tables

Supplementary Figure legends

Figure S1. (A) Alignment of BGIBMGA012088 and orthologs among several insect species. The red line represents the Pyridoxal Phosphate domain. The protein sequences used for Alignment were listed as follow: *Danaus plexippus* EHJ66406.1, *Bombyx mori* BGIBMGA012088, *Biston betularia* AEP43793.2, *Papilio xuthus* BAI87832.1, Bicyclus anynana AEQ77286.1, *Manduca sexta* Msex2.11697-RA, *Tribolium castaneum* ABX89951.1, *Drosophila melanogaster* NP_476788.1. (B) A neighbor-joining phylogenetic tree of amino acid decarboxylase with Pyridoxal Phosphate domain based on their amino acid sequences, with bootstrap values from 1000 replications. The numbers on the branches represent the bootstrap values. Bm=*Bombyx mori*, Dm=*Drosophila melanogaster*, Tc=*Tribolium castaneum*. (C) Predicted tertiary structure of BmADC between wild-type and *bp* mutant.

Figure S2. Phenotype of Dazao (wild-type) and 16-100 (*bp* mutant) at larvae and moth stage. (A) Phenotype of Dazao and 16-100 on day 3 of the 5th instar larvae. a, b represent the dorsal side phenotype of wide-type (Dazao) on day 3 of the 5th instar larvae, respectively; c, d represent the ventral side phenotype of 16-100 day 3 of the 5th instar larvae, respectively. Scale bar: 1 cm. (B) Phenotype of Dazao and 16-100 at the first day of the moth stage. a, b represent the dorsal side phenotype of Dazao and 16-100 at the first day of the moth stage, respectively; c, d represent the ventral side phenotype of Dazao and 16-100 at the first day of the moth stage, respectively. Scale bar: 1 cm.

Figure S3. The retention times of amino acids and catecholamine standards. (A) The retention times of

Aspartate and β-alanine standards (aspartate 11.76 min；β-alanine, 59.27 min). (B) The retention times of Dopamine and NBAD standards (Dopamine, 8.065 min; NBAD, 18.404 min).

Figure S4. Expression patterns of β-alanine synthesis and melanin metabolism genes during several developmental stages. (A) In Dazao strain, Expression patterns of β-alanine pathway rate-limiting enzyme genes, *BmADC* and *BmDYPD*, during different development stages. 4M: the 4th molting stage; V: the 5th instar larvae stage; W: the wandering stage; P: the pupal stage; M0: day 1 of themoth stages. (B) In Dazao strain, the relative expression level of Bmebony and BmaaNAT (both of them contribute to Dopamine consumption) during the pupal-adult stage. P: pupal stage; M0: day 1 of the moth stages.

Figure S5. The pupae color pattern and the relative expression levels of *BmADC* between Dazao and 16-100 in different temperature conditions. (A) The schematic diagram of HSF (Heating shock factor) binding site on upstream sequence (~6.3 kb) of the transcriptional start site of *BmADC*. The red boxes represent HSF binding sites. The green box represents the SINE-like insertion in the bp mutant. (B) a, b, c represent the dorsal side phenotype of Dazao at 6 h of pupation three different temperature conditions (20℃,24℃and 30℃). d, e, f represents the dorsal side phenotype of 16-100 at 6 h of pupation in three different temperature conditions (20℃, 24℃and 30℃). Scale bar: 1 cm. (C) Relative expression levels of *BmADC* between Dazao and 16-100 at 6 h of pupation in different temperatures (20℃,24℃and 30℃). (Student's t-test, n=3, **, p<0.01. Data are presented as mean±SD).

Figure S1

A

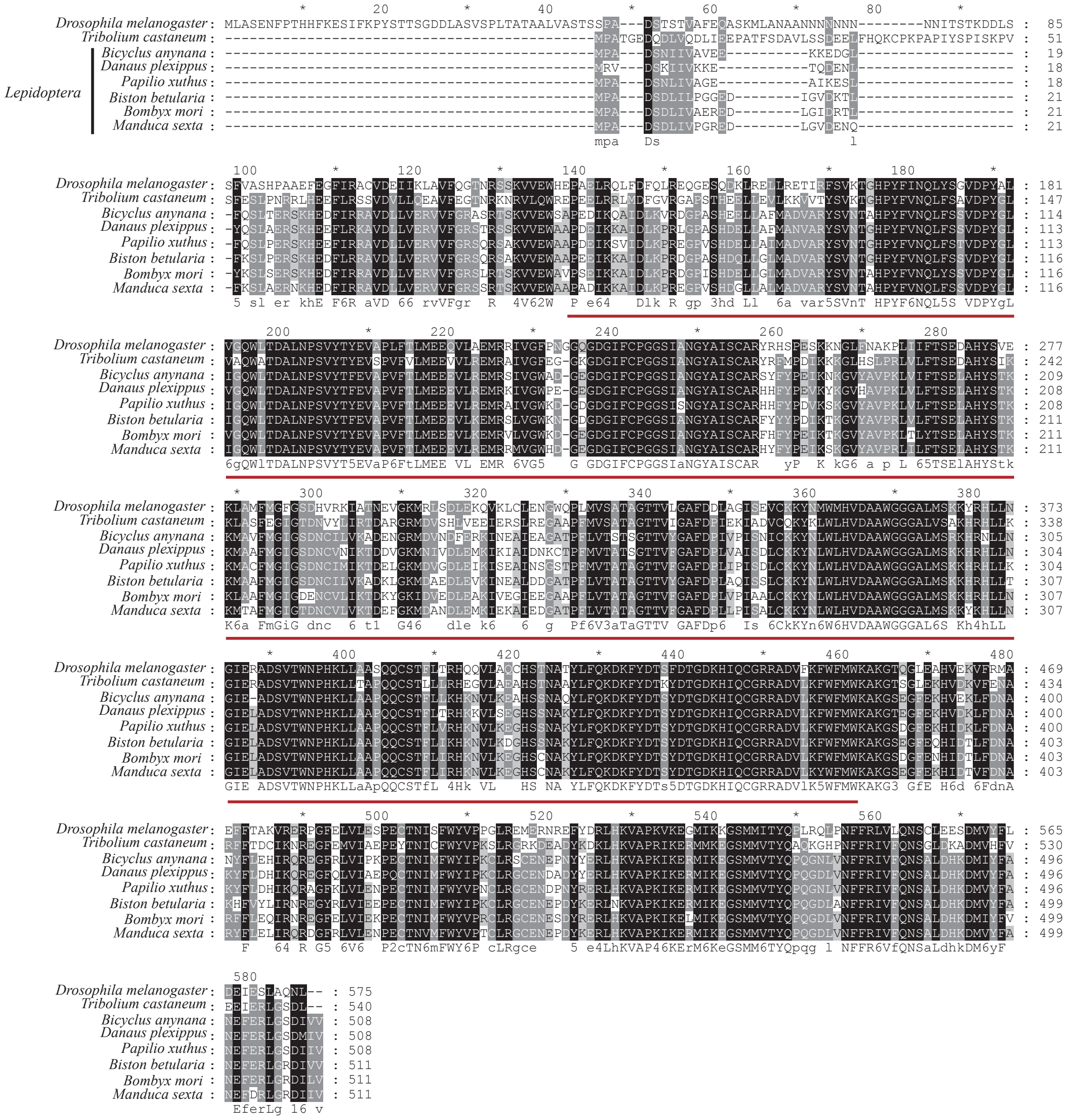

B

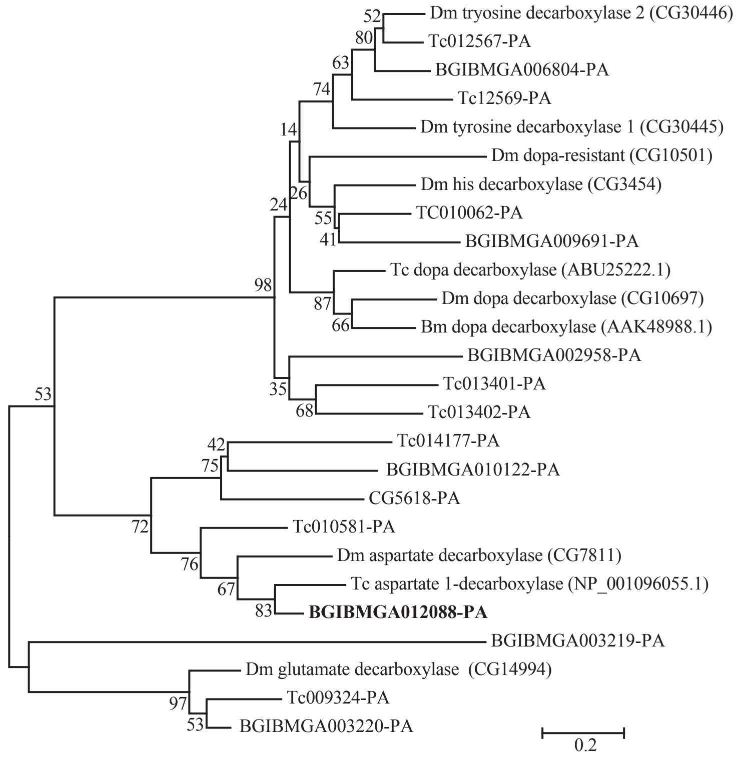

C

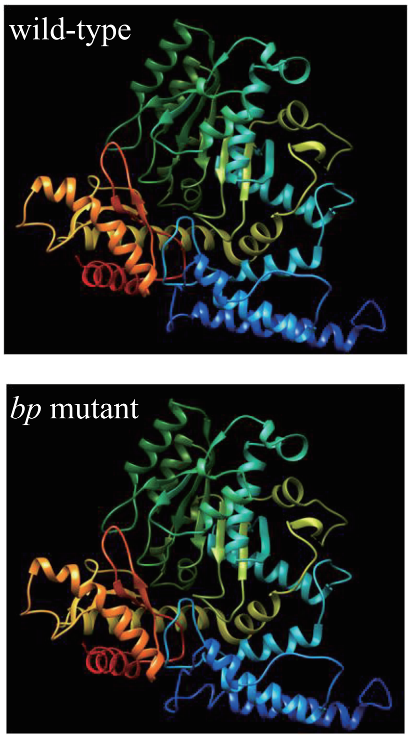

Figure S2

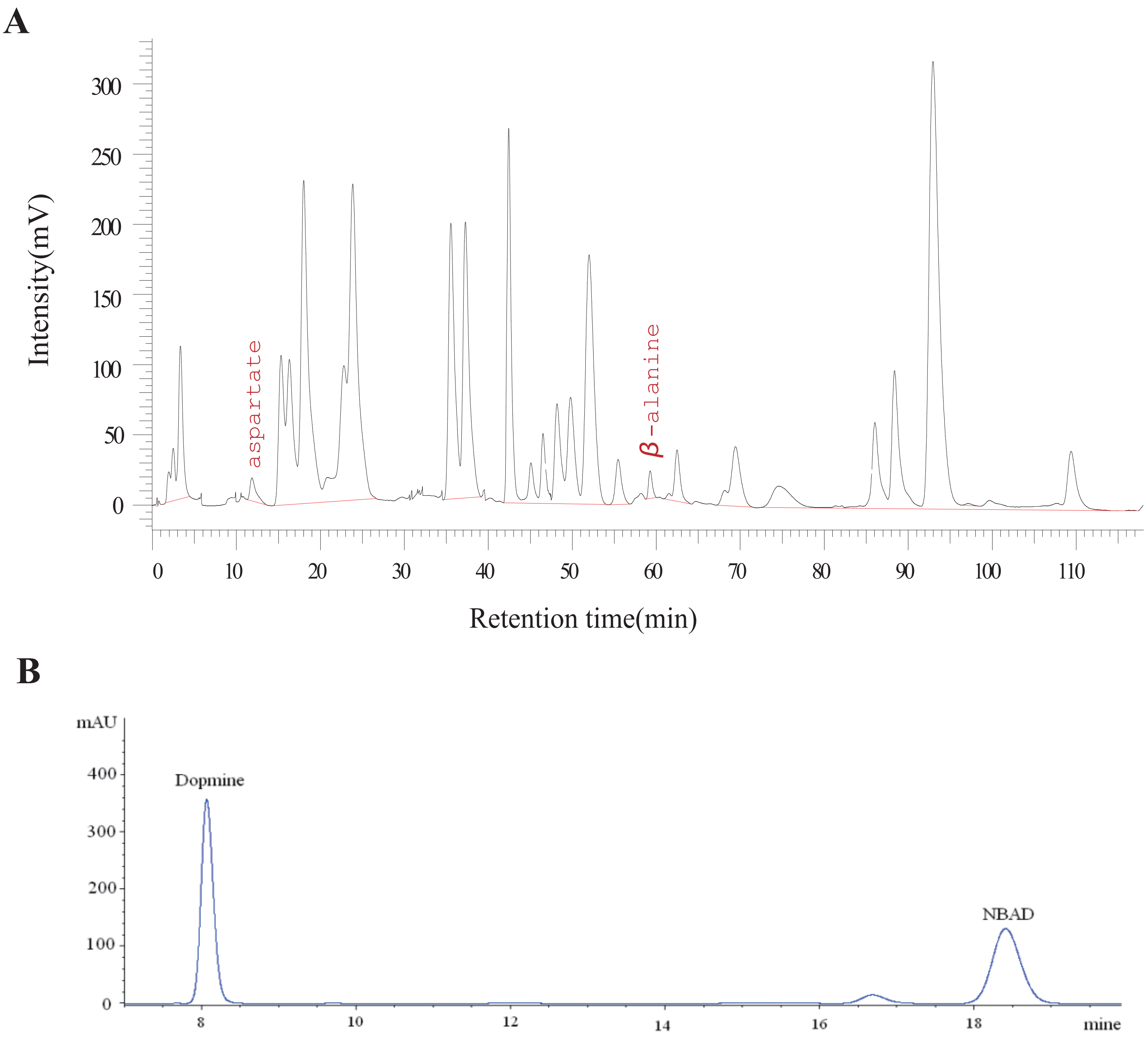
A
Intensity(mV)
300
250
200
150
100
50
0
aspartate
β-alanine
0
10
20
30
40
50
60
70
80
90
100
110
Retention time(min)
B
mAU
400
300
200
100
0
Dopmine
NBAD
8
10
12
14
16
18
mine

Figure S3

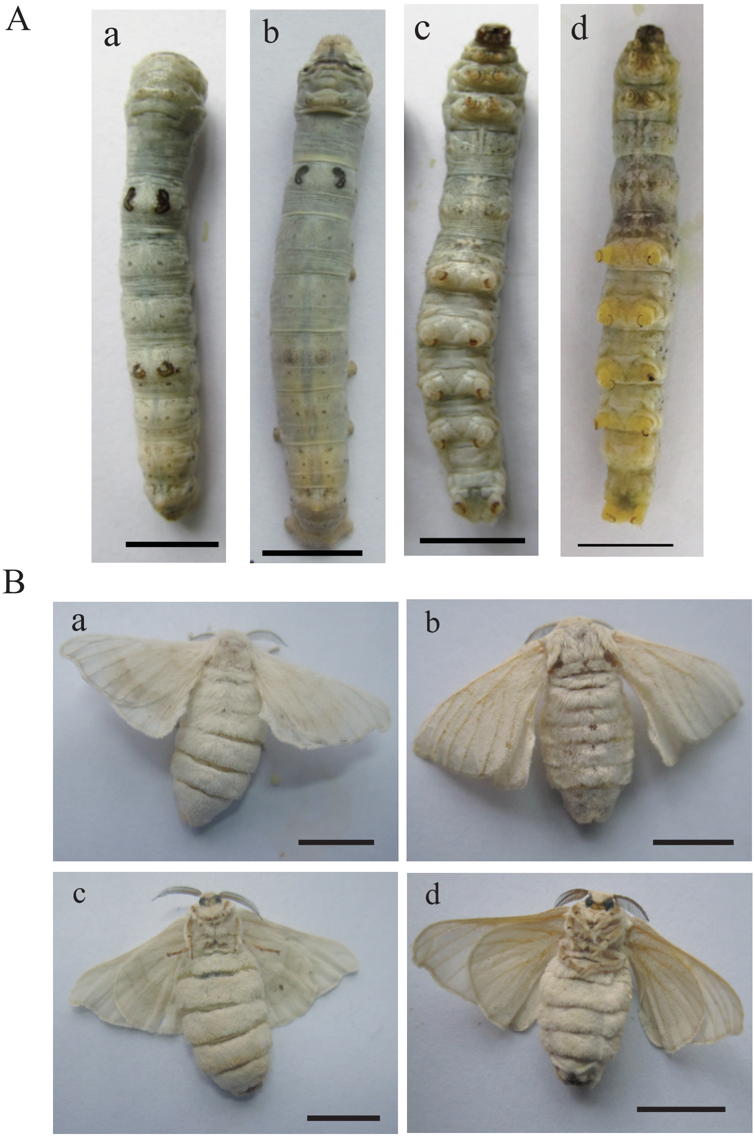

Figure S4

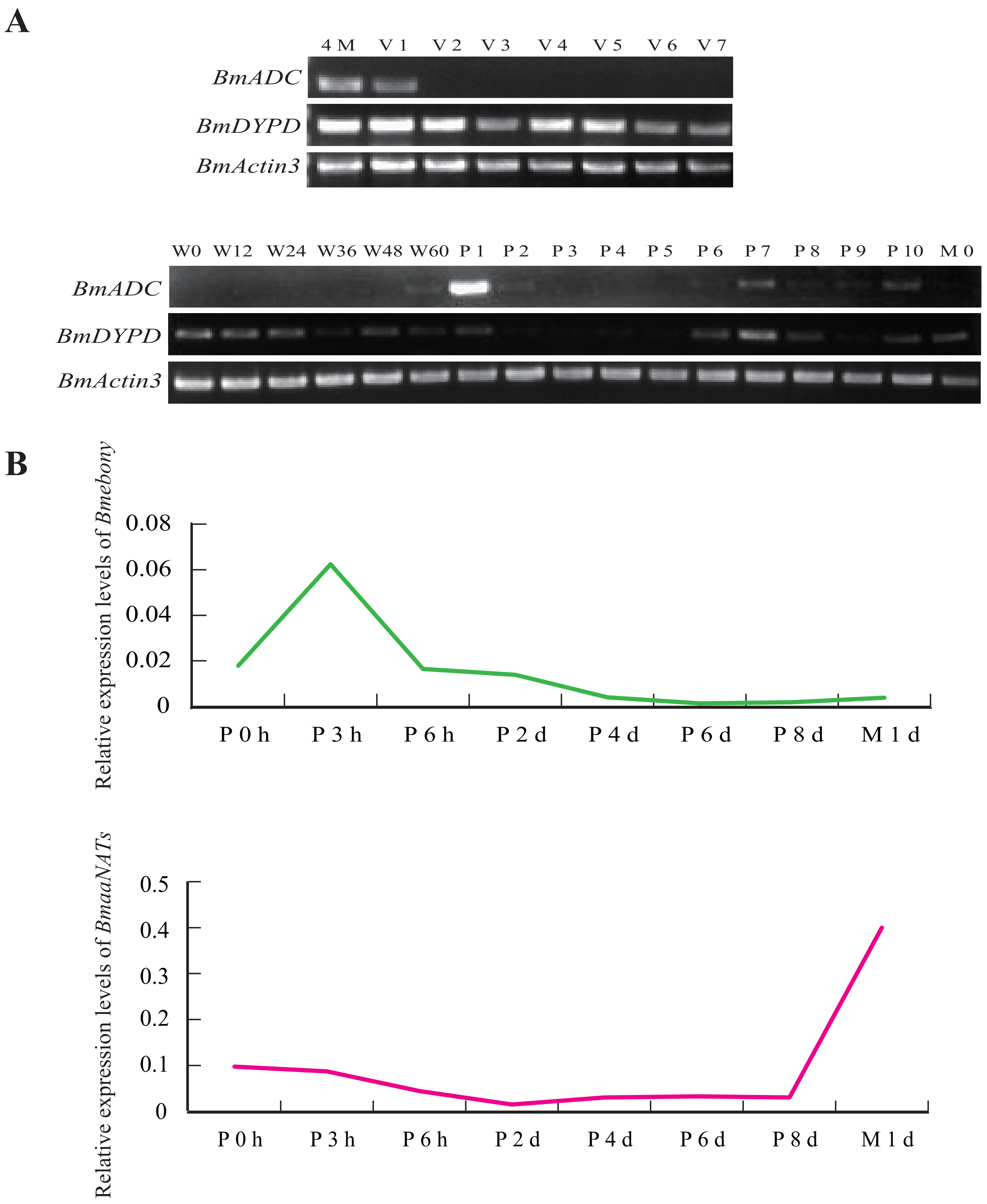
A
4 M V 1 V 2 V 3 V 4 V 5 V 6 V 7
BmADC
BmDYPD
BmActin3
W0 W12 W24 W36 W48 W60 P 1 P 2 P 3 P 4 P 5 P 6 P 7 P 8 P 9 P 10 M 0
BmADC
BmDYPD
BmActin3
B
Relative expression levels of *Bmebony*
0.08
0.06
0.04
0.02
0
P 0 h P 3 h P 6 h P 2 d P 4 d P 6 d P 8 d M 1 d
Relative expression levels of *BmaaNATs*
0.5
0.4
0.3
0.2
0.1
0
P 0 h P 3 h P 6 h P 2 d P 4 d P 6 d P 8 d M 1 d

Figure S5

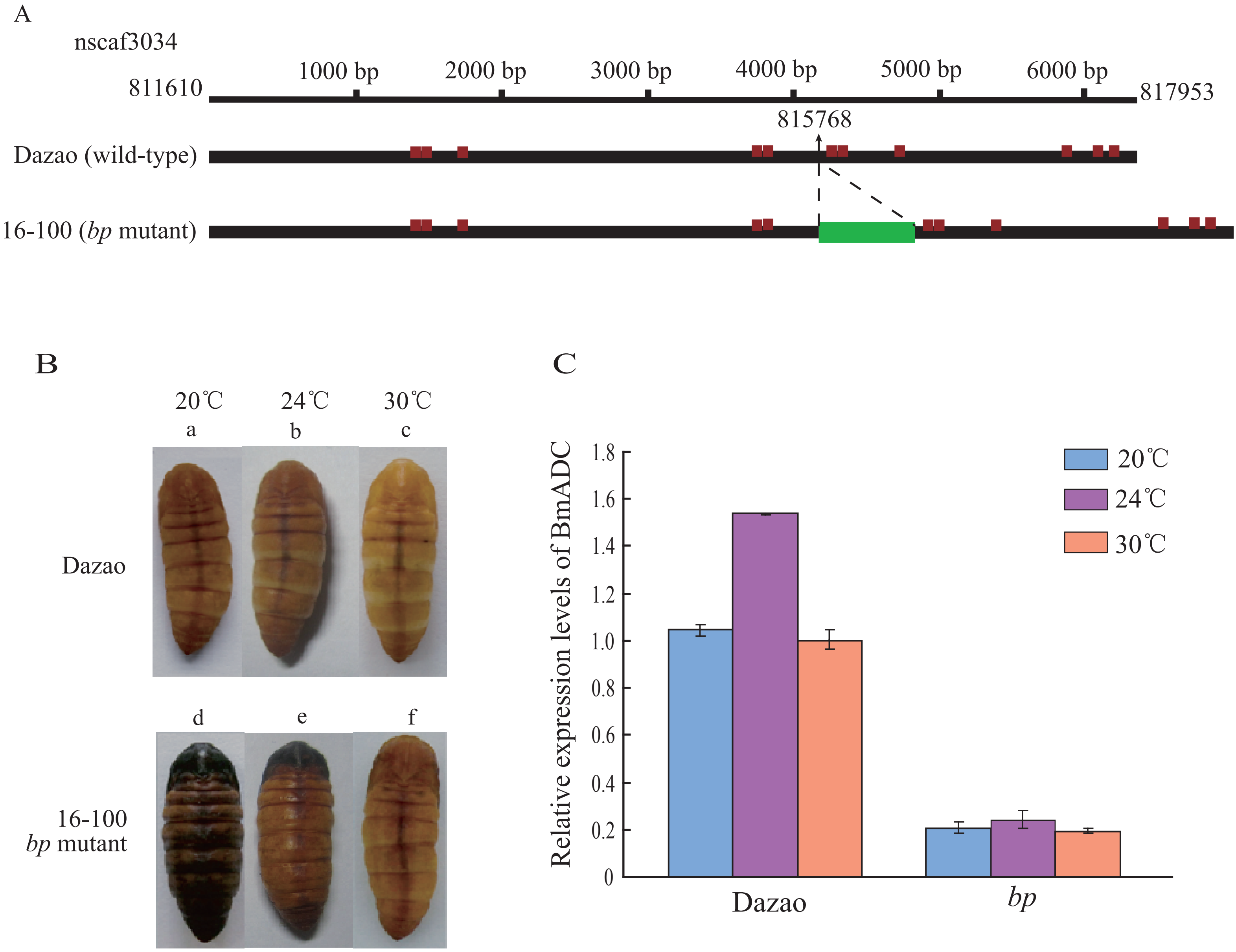

A
nscaf3034
811610
1000 bp
2000 bp
3000 bp
4000 bp
5000 bp
6000 bp
817953
815768
Dazao (wild-type)
16-100 (bp mutant)
B
20℃
24℃
30℃
a
b
c
Dazao
d
e
f
16-100
bp mutant
C
Relative expression levels of BmADC
1.8
1.6
1.4
1.2
1.0
0.8
0.6
0.4
0.2
0
20℃
24℃
30℃
Dazao
bp

Table S1. Primers used in this study.

Primer name	Sense (5'-3')	Anti-sense (5'-3')
mapping S1101	CGACTCACTCCTTTTATTTTATTGACTCT	CGGATCGAGTACTGCAATGCG
mapping S1102	AAAAGAAATGGCTGAACCAATAA	GGAGTTAGAACGAACGCCACG
mapping S1103	ATCTTCCCCTTCGTCGTTATA	GCGTGCTGATGCATGGGTAAC
mapping S1104	GTCCCAAATGTTGTTGTTCGG	CGTTAGGGTGAAGCTGAAATAGG
mapping S1105	TTAGATAGGCAGACAACGGCG	TTGCCATTTCACCGTCCG
mapping S1106	GCCGTGCTGGACATAGGACTT	CCAGTCATCTTGGAATGCGTTT
mapping S1107	GTCCGCTAAATACTTATTGTTACGAAA	TTTGAAGGGATTTCCATTAGCG
mapping S1108	TTGAAGATAGAGCGAAGTGGAGG	GCGGCAGAAATAGGACGGT
mapping S1109	CAAATATCCGTAAAGTCCCCAG	CGACCCTACCTAAAGTGGGATAAGA
mapping S1110	CGGTGGCAGAAGGTCTCATC	TCATGAGGTCCGGAATTTAAGTAA
mapping S1111	AACCTGTGAGCTGACCTAACCG	AACAAGATAGTCCGCAACCCAG
mapping S1112	TATGCCAAGGTGTTCGACGG	AATTTGCTCATAAACCCGCG
mapping S1113	GGAGTAAAATCGCCGCCATA	AACTCCTTCGACATCCTCCGTAC
mapping S1114	TCCTAGTTCCGACCCCGTATCT	CGATTATAGAACTTTAGCAGAAGTGTATCT
mapping S1115	CCCTCAACCAGATGCAGCGA	ACCGTTATAGATGCTAAAGATATGATTGT
mapping S1116	TTTTAGTGTAGGGCACAATTCCAA	TAGCGATAAGACCGCCTATTGTAC
mapping S1117	GCCACCAGTAAAAGCCTGAAGA	GGGTTGTTGTTAGACAGGCGG
mapping S1118	ACGGCAGCACACGCATAAAAC	GCACTAGTGTCACGACGGCAATC
mapping S1119	AAAACACGGTGGTGCAACGC	CTA CAA AAT ACC ATT ACT GAC ACC GA
mapping S1120	CTTCACTGCTCGCGTTCCC	GAGGCTTTCGTGCCAATGAG
mapping S1121	GGAGAGGTGTTAGGTTGTTTTCGT	CAGCAGCGAGCGGTCCC
mapping S1122	TCTCATACCTGGCTTACCACGTT	GAAATGGTAGTGCAAGGTAGAGGG
mapping S1123	5'-GATTTACCATCTAATTCAACACCG-3'	5'-TGCAGTTACTCGCTCCTCG-3'
mapping S1124	CGGACAGCTATAAATTGTACCATCG	CGATAAGACCGCCTATTGCAC
mapping S1125	CAGAATCAAATACGTGCCTCAGTT	GGAGGAGCGTTTGCGTGTTA
mapping S1126	GGAATAAGGTCGCCAAAGGTG	CCACTGCACCAGACACGACTG
mapping S1127	AAATTTCATACTCCTCCGTCCG	CATTTCTACCACAGCCAAACGAT
mapping S1128	CACGCCTTGAAGTTGATTGGAT	GCGTGGTATGGGTATGTGATGC
mapping S1129	TTTATCGCCAATACCAGTCCAA	GATAGATTAGATTGGGTTAAGTCGTGA
mapping S1130	TGCGGGAGGCTGGGG	CACGACGCACGTAGTTTCCG
mapping S1131	TTGATAGAAGAGGGATGAAGCAGTT	TTGAGACTATCGCCAACTGCC
mapping S1132	ACATTACTCAGTTTCGCTGTTCGT	GGACATCGCCAAGGGACG
mapping S1133	CGGTCCTTTGCTTTAGCGATA	CAGCGGTCCGGTTTCAGTTT
mapping S1134	ATGACACGGTGCGACGAAA	CCCGTGTCCATAGATGAAGCG
mapping S1135	CGGCTTTGGCTATTGTTTACTTAC	CCAGTTTACATGACCCAAATTTAGAT
mapping S1136	GATTTCCATCAGAGTAACGGGTG	TCTGCATTTGGATATCACGGAG
mapping S1137	TTGCTCTGAGCGATAAGACTGC	TGAGCCTGTATTCCATGAAAACG
mapping S1138	CCCAGAGTGAGACTGCTCCATT	TTGTTACTCACTGAGGGTCGGG
mapping S1139	TGGTGGCGTAGAAGCCTTGTA	GAAACGCTTGACTGGAAGAGGT
mapping S1140	GCCACAAATAAAGATTACCGTAGCTC	TGCGTGAAAGCCGATATGTG
mapping M1	CCACATCATTGTTATGCAGTCT	AAGGTTAAGTGCGTAAGGGTAT
mapping M2	ACGGCGTCACCTTTGCTTT	AGCCGAGAAACTCAGCAGGT
mapping M3	GTTGGCGTTACTTATCCGTGTT	GTGCTGGCTACCTGACCTG
mapping M4	TGGGCTTTCAAGTCCTGG	TGCCTCTATTTGCTTACTGATG
ADC-ORF	AAGTAGATCACGATGCCTG	AGATGCGAGATTTTTTGTA
5'-ADC	CGACTGGAGCACGAGGACACTGA	CCATACGGGTCCACGGATGAATAC
5'Nested-ADC	GGACACTGACATGGACTGAAGGAGTA	CGAACACTACCCTTTCAACCAGCA
3'-ADC	GCTGTCAACGATACGCTACGTAACG	ACGACGGGCGGATGTCCTCAAAT
3' Nested -ADC	CGCTACGTAACGGCATGACAGTG	TCCCGATGGTAACTTATCAGCCTCA
RT-PCR-BmADC	GCATCAGTTGTATTCATCCGTGG	TCGCTATGGGGACTAATGGGT
RT-PCR-BmDYPD	CGAGAACTACGGTCCAGGTCA	CTTCATAAGCAGCCACAGCG
BmActin3	CATGAAGATCCTCACCGAGCG	CGTAGCACAGCTTCTCCTTGATA
qRT-BmADC	GTGGGCGGATGTCCTCAAA	GCAAGCCTTCCCGATTACG
qRT-BmDDC	AGCCTTGGACTGCGGTGAT	ATAGCGGGATACGAGTTAGCG
qRT-Bmebony	GACGCCCAGGTCAAGATTCG	CGTAGCATAGCACCACGCACT
sw22934	TTCGTACTGCTCTTCTCGT	CAAAGTTGATAGCAATTCCCT
dsBmADC1	TAATACGACTCACTATAGGGAGAATTGCCAACGGTTATGCCA	TAATACGACTCACTATAGGGAGACGAAGCCTTCCCGATTACG
dsBmADC2	TAATACGACTCACTATAGGGAGAACCCTAATGGAGGAAGAAGTGC	TAATACGACTCACTATAGGGAGAGTGGAGCATTGTTGAGGAGC
dsRed	TAATACGACTCACTATAGGGAGACTTCAAGGTGCGCATGGAG	TAATACGACTCACTATAGGGAGATGTGGATCTCGCCCTTCAG

Table S2. Statistic of RNAi

Treatment	Injected	Death	Pupation	Melanism	Efficiency
dsBmADC	40	10	30	17	56.7%
dsRed	23	5	18	0	0%

Table S3. Statistic of β-alanine treatment

Treatment	Injected	Death	Pupation	Revert to wild-type	Efficiency
β-alanine	72	9	63	42	66.7%
saline	24	3	21	0	0%
